# Antimicrobial resistance genes and antibiotic use in chronic lung disease: a bronchoscopy study of the lower airways microbiome

**DOI:** 10.1136/bmjresp-2025-003864

**Published:** 2026-03-25

**Authors:** Guri D. Kringeland, Solveig Tangedal, Daniel Julian, Andreu Paytuví-Gallart, Walter Sanseverino, Randi J. Bertelsen, Gunnar R. Husebø, Kristel S. Knudsen, Sverre Lehmann, Rune Nielsen, Tomas M. L. Eagan

**Affiliations:** 1 Department of Clinical Science, Faculty of Medicine, University of Bergen, Bergen, Norway; 2 Department of Thoracic Medicine, Haukeland University Hospital, Bergen, Vestland, Norway; 3 Sequentia Biotech SL, Barcelona, Catalonia, Spain

**Keywords:** COPD Pathology, Idiopathic Pulmonary Fibrosis, Sarcoidosis, Bacterial Infection, Microbiome

## Abstract

**Background:**

Antimicrobial resistance genes (ARGs) in the respiratory microbiome are poorly characterised. We compared the presence of ARGs in healthy controls with patients with chronic lung disease in a cross-sectional study, adjusted for time since antibiotic use.

**Methods:**

Bronchoalveolar lavage was collected from 100 controls, and 93 patients with chronic obstructive pulmonary disease (COPD), 13 with asthma, 34 with sarcoidosis, 12 with idiopathic pulmonary fibrosis (IPF) and 11 patients with unclassifiable interstitial lung disease (uILD). Participants had not used antibiotics 14 days prior to sampling. Shotgun metagenomic sequencing was performed with Illumina NovaSeq. ARGs were identified using the National Database of Antibiotic-Resistant Organisms. Sample reads were normalised to counts per million.

**Results:**

In total, 38% of controls had at least one ARG, compared with 51%, 39%, 65% and 83% of patients with COPD, asthma, sarcoidosis and IPF, respectively (p=0.01). ARGs against tetracycline (33%) were the most common ARG class, followed by beta-lactam and macrolide resistance (both 26%). In a logistic regression analysis adjusted for sex, age, body composition, smoking and antibiotic use, the OR (95% CI) for having ARGs in the lower airways was 1.30 (0.70 to 2.41) in COPD, 1.00 (0.29 to 3.52) in asthma, 3.52 (1.40 to 8.83) in sarcoidosis, 6.40 (1.25 to 32.73) in IPF and 3.27 (0.76 to 14.16) in uILD compared with controls. Overall mean (SD) ARG counts per million were 403.8 (537.7) in the 35 subjects who had used antibiotics ≤3 months before bronchoscopy, compared with 197.6 (355.9) in the 228 subjects without (p=0.02).

**Conclusion:**

The presence of ARGs in the lower airways microbiome was significantly higher in patients with sarcoidosis and IPF than in controls. The counts per million for ARGs were significantly associated with recent antibiotic use.

**WHAT IS ALREADY KNOWN ON THIS TOPIC**

- The prevalence of antimicrobial resistance genes in the lower airways is not well known in health or chronic lung disease. Patients with chronic lung disease have a dysbiosis in the respiratory microbiome and frequently use antibiotics for respiratory infections.

**WHAT THIS STUDY ADDS**

- This study shows that patients with chronic lung diseases have higher prevalences and higher burden of antimicrobial resistance genes in their microbiomes. Antibiotic use was one factor which most closely correlated with presence of antibiotic resistance genes.

**HOW THIS STUDY MIGHT AFFECT RESEARCH, PRACTICE OR POLICY**

- Overuse of antibiotics for respiratory infections might select for more resistance genes, detrimental to individual patients later in their course of disease and perhaps represent a source of lateral transfer of resistance in the population.

## Introduction

 Dysbiosis can be defined as an imbalance in the composition, diversity or function of microbial communities. Studies have found differences in the microbial composition of the lower airways between healthy subjects and patients with different chronic lung diseases like cystic fibrosis,^1^ chronic obstructive pulmonary disease (COPD),^2^,^7^ idiopathic pulmonary fibrosis (IPF),^8 9^ chronic hypersensitivity pneumonitis^10^ and sarcoidosis.^11^ Typically, these studies have used amplicon sequencing, most often 16S rRNA of sputum samples, when characterising the lower airways bacterial microbiome.

Studies on functional dysbiosis in the respiratory microbiome are few. One important class of functional genes is antimicrobial resistance genes (ARGs), and the term resistome is often used for the composition of ARGs in a microbiome. For millions of years, microbes have produced substances with antimicrobial properties as part of the natural competition between different microbes in a microbial ecosystem. Most antibiotics have come from harvesting this natural resource, like penicillin. When we use antibiotics in food production or against diseases, a selective evolutionary pressure is applied, allowing microorganisms with either inherent or newly acquired ARGs to survive and proliferate.^12^ Bacteria can pass on ARGs vertically through inheritance from parents to offspring, or horizontally through lateral gene transfer, which can occur also between different strains and genera.

Widespread antimicrobial resistance (AMR) is fast becoming one of the greatest health challenges in the world today.^13^ New drug development alone is unlikely to solve this, as microbes rapidly develop resistance. Understanding resistance dynamics is vital to enable better use of existing drugs.

Knowledge of the prevalence of ARGs in disease and health is nearly non-existent for the lower airways microbiome.^14^ Two previous studies have characterised the resistome in patients in intensive care units,^15 16^ two studies have examined sputum samples from patients with bronchiectasis^17 18^ and one study the resistome in COPD.^19^ The number of healthy subjects from all these studies combined is 73, and most samples are sputum from the upper airways. Only the study by Ramsheh *et al* examined previous use of antibiotics, in a subcohort of healthy subjects.^19^

In the single centre MicroCOPD and MicroILD studies from Western Norway, more than 200 subjects with COPD or different interstitial lung diseases (ILDs), as well as over 100 healthy controls, were examined by bronchoscopy with protected, sterile sampling from the lower airways.^20^ With amplicon sequencing, we have previously shown that there is a dysbiosis in COPD,^7^ sarcoidosis^11^ and IPF.^8^

We performed shotgun metagenomic next-generation sequencing (mNGS) on bronchoalveolar lavage (BAL) samples from the two studies, to assess the presence and burden of ARGs in controls and in patients with COPD, asthma, sarcoidosis, IPF and unclassifiable ILD (uILD). Further, we examined whether previous use of antibiotics influenced the burden of ARGs.

## Methods

### Study population

Data collection in the MicroCOPD and MicroILD studies was performed between 2012–2016. Both study protocols have been published.^8 20^ All participants were enrolled from the outpatient clinic at the Department of Thoracic Medicine, Haukeland University Hospital, Bergen, Norway. Participants from both studies were examined at the same bronchoscopy laboratory by the same study team, and with the same equipment and sample collection methods and handling.

The MicroCOPD study included 130 patients with COPD, 16 patients with asthma and 103 healthy controls. The MicroILD study included 35 patients with sarcoidosis and 33 patients with suspected ILDs, of which 12 were later confirmed to have IPF after a full diagnostic work-up.^21^ 11 patients with ILD remained categorised as uILD.

A patient history was obtained through a medical interview by one of the study physicians, using a standardised questionnaire, which included comorbidities, medication use including prior antibiotic usage the last year, smoking habits and exacerbation rates in case of COPD. Bioelectrical impedance measurements were made with a Bodystat 1500 for the characterisation of body composition.^22^ Forced expiratory volume in 1 s (FEV_1_) was measured post-bronchodilatation and used to categorise patients with COPD by Global Initiative for Chronic Obstructive Lung Disease stages.

Participants could not be included if they had used antibiotics the previous 14 days, whether prescribed for lung disease or for other indications. Long-term macrolide treatment was cause for non-inclusion. BAL sampling was not performed in patients with COPD with both FEV_1_ <1 L and <30% of predicted. The current analysis includes only participants with a valid BAL sample. These inclusion criteria left us with 100 controls, 93 patients with COPD, 12 patients with asthma, 35 patients with sarcoidosis, 12 patients with IPF and 11 patients with uILD (online supplemental figure 1).

### Sample collection

Bronchoscopy was performed with the participants in the supine position through oral access. Intravenous sedative (alfentanil 0.25–1 mg) was offered, and lidocaine was applied through the bronchoscope. To minimise contamination from the upper respiratory tract, no suction was performed before entering below the vocal cords. BAL was always sampled from the middle lobe, with two or three aliquots of 50 mL phosphate-buffered sterile saline (PBS) through a sterile protective sheet inserted through the bronchoscope. A negative control sample was collected from the PBS bottle for each participant.^20^ All samples were aliquoted in 2 mL vials and stored immediately in −80°C freezers until use.

### DNA extraction and sequencing

DNA extraction of the BAL samples was performed as previously described.^23^ The DNA extraction was performed between 2016 and 2018 in the larger MicroCOPD/MicroILD cohort and stored in two aliquots per sample until sequencing. DNA input concentrations were quantified using the Qubit V.2.0 Fluorometer (Invitrogen, Carlsbad, California, USA). A subset of samples was re-extracted in 2021, due to low amount of DNA from the first extraction. This was also the case for all the selected negative control samples, from which DNA yield remained too low for sequencing, also after the second round of extraction. Storage time at −80°C thus varied between 3 and 6 years for the samples in the current study cohort.

Library preparation was performed using the Celero DNA-Seq library preparation kit (Tecan, Männedorf, Switzerland) following the manufacturer’s PCR-based workflow, which is recommended for low-input samples. Library amplification was carried out using 9–11 PCR cycles, depending on the amount of input DNA. Final libraries were assessed for quantity using Qubit V.2.0 fluorometer, and for fragment size distribution and quality using the Agilent 2100 Bioanalyzer High Sensitivity DNA assay (Agilent Technologies, Santa Clara, California, USA). Libraries meeting quality criteria were subjected to shotgun metagenomic sequencing performed on an Illumina NovaSeq 6000 in paired-end 150 bp mode.

### Bioinformatics

The sequencing data initially underwent a quality check and trimming step applying BBDuk^24^ with a minimum Phred quality score of 25 and minimum length of 35 base pairs. The high-quality sequencing reads were mapped with Bowtie2^25^ against the human genome version hg38 to identify and discard reads derived from the host. After removal of reads derived from humans, the average reads per sample was 1.6 million. 94.9% of reads had a minimum Phred quality score of 25 (and 81.1% of reads had a Phred score ≥30%).

Non-human derived reads were then aligned against a protein database containing sequences from the UniRef90^26^ and National Database of Antibiotic Resistant Organisms (NDARO), National Center for Biotechnology Information (NCBI) databases. The database was chosen due to its larger number and diversity of AMR genes, regular update frequency and high curation quality.^27^ A detailed description of a comparison between NDARO and the Comprehensive Antibiotic Resistance Database is provided in the online supplemental Text. Protein alignments were carried out using DIAMOND blastp^28^ setting thresholds of ≥80% alignment identity and ≥90% read alignment coverage. The DIAMOND output was processed using the FAMLI tool,^29^ which evaluates read depth consistency across protein sequences to reduce false positives. Only proteins with a depth SD-to-mean ratio <1 and with ≥70% of their length covered by aligned reads were retained for downstream analysis.

### Statistical analyses

All reads assigned to a protein, independently of whether an ARG or not, were normalised using counts per million to adjust for differences in sequencing depth. ARGs were grouped by AMR class and subclass. The presence or absence of ARGs was analysed as percentages and compared between groups using χ^2^ test. Logistic regression models were fitted with sex, age, body composition, smoking and use of antibiotics as predictor variables, and presence of any ARG as outcome. Differences in burden of ARGs, by number of count-per-million reads between study categories, were analysed with Kruskal-Wallis tests. Linear regression models were fitted with the geometric mean (GM) ratio of number of counts per million as outcome and the same predictor variables as above to assess differences in burden of ARGs between groups. The statistical analyses were performed with Stata SE V.18 and a p value of <0.05 deemed significant.

### Patient and public involvement statement

When the study was planned in 2012, no structured patient or public involvement was included. However, as the study evolved, we formed two participant advisory groups. We consulted the groups on our information strategy for the results from the study, as well as sought advice on inclusion and information procedures in future studies.

### Role of the funding source

The data collection in this study was supported by unrestricted research grants from the Helse-Vest regional health authorities in 2014–2015.

The mNGS analyses were partially funded by GSK under the Investigator Supported Studies programme with grant number 214 165. GSK was provided the opportunity to review a preliminary version of this publication for factual accuracy, but the authors are solely responsible for final content and interpretation.

Sequentia Biotech helped with the bioinformatics analyses, particularly identifying the ARGs from the mNGS sequences, their work hours were partially financed by the European Innovation Council, grant agreement number 190 195 702.

## Results

### The study population

The majority of patients with sarcoidosis and IPF were male. The patients with IPF and uILD were more likely to have used antibiotics within 3 months prior to inclusion. In contrast, patients with sarcoidosis were less likely to have used antibiotics the previous year. Lung function varied among the study groups, with patients with COPD having lower FEV_1_ while patients with IPF exhibited the most pronounced decreased diffusing capacity of the lungs for carbon monoxide (D_L_CO) (online supplemental table 1).

### The presence of ARGs

ARGs were detected across all study categories, including controls, but the prevalence was significantly higher in patients with sarcoidosis, IPF and uILD (table 1). Although the prevalence of ARGs was higher among patients with COPD than controls, the difference was not statistically significant.

**Table 1:** Presence (%) of any antibiotic resistance genes in bronchoalveolar lavage, by study category

	Controls	COPD	Asthma	Sarcoidosis	IPF	uILD	Ctrl versus diseases*
	n=100	n=93	n=13	n=34	n=12	n=11	
Tetracycline class							* † ‡
No	76.0	65.6	84.6	55.9	25.0	45.5	
Yes	24.0	34.4	15.4	44.1	75.0	54.5	
Beta-lactam class							§ ¶
No	80.0	77.4	84.6	52.9	50.0	63.6	
Yes	20.0	22.6	15.4	47.1	50.0	36.4	
Cephalosporin subclass							
No	96.0	95.7	100	100	91.7	90.9	
Yes	4.0	4.0	0.0	0.0	8.3	9.1	
Carbapenem subclass							
No	99.0	97.8	100	94.1	100	90.9	
Yes	1.0	2.2	0.0	5.9	0.0	9.1	
Macrolide class							* †
No	83.0	73.1	84.6	64.7	33.3	54.5	
Yes	17.0	26.9	15.4	35.3	66.7	45.5	
Macrolide-streptogramin class							†
No	87.0	77.4	92.3	73.5	50.0	72.7	
Yes	13.0	22.6	7.7	26.5	50.0	27.3	
Lincosamid-macrolide-streptogramin class							‡
No	97.0	91.4	92.3	88.2	83.3	72.7	
Yes	3.0	8.6	7.7	11.8	16.7	27.3	
Aminoglycoside class							
No	98.0	95.7	92.3	100	91.7	100	
Yes	2.0	4.3	7.7	0.0	8.3	0.0	
Any ARG mechanism							* † ‡
No	62.0	49.5	61.5	35.3	16.7	27.3	
Yes	38.0	50.5	38.5	64.7	83.3	72.7	

* χ2 test. In cases of groups <5, Fisher’s exact test was used. Ctrl versus sarcoid.

† For ctrl versus uILD.

‡ Where *p<0.05 and *p<0.01. For ctrl versus COPD or asthma, there were no statistically significant differences.

§ For ctrl versus IPF.

¶ For ARG classes fosfomycin, lincosamide, nitromidazole, phenicol, lincosamide-streptogramin, quinolone, sulfonamide and trimethoprim, only between one and five positive instances were found and these are not included in the table.

ARG, antimicrobial resistance gene; COPD, chronic obstructive pulmonary disease; ctrl, control; IPF, idiopathic pulmonary fibrosis; uILD, unclassifiable interstitial lung diseases.

**Figure 1:**
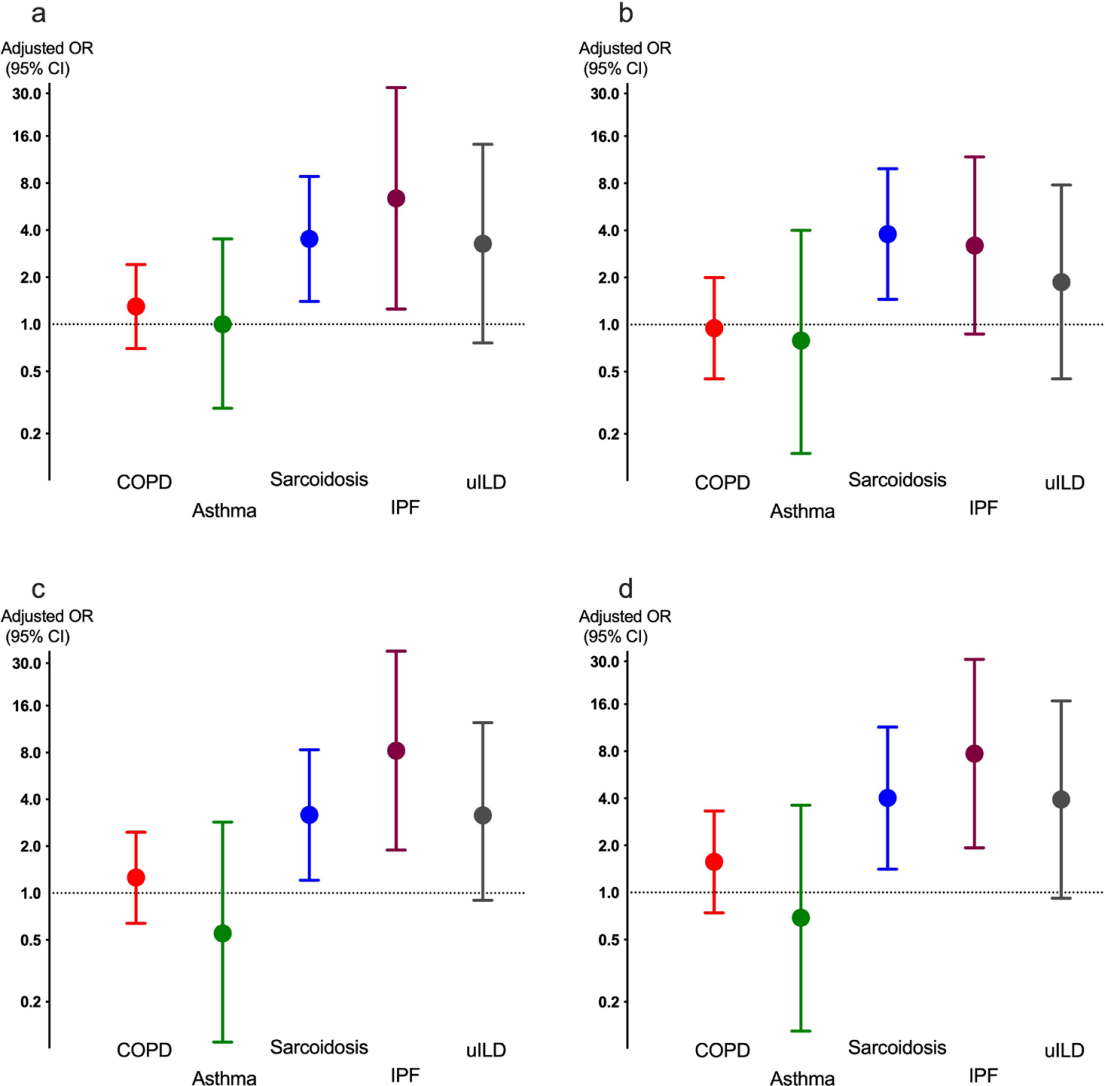
The OR (95% CI) for having (a) any (b) at least one beta-lactam (c) at least one tetracycline, and (d) at least one macrolide resistance mechanism found in the respiratory microbiome in patients from each of the four diagnoses compared with healthy adults; adjusted for sex, age, body composition, smoking and use of antibiotics in the last year. COPD, chronic obstructive pulmonary disease; IPF, idiopathic pulmonary fibrosis.

When classified by AMR class, tetracycline resistance mechanisms were most common, beta-lactam resistance second and macrolide resistance third (figure 1).

No significant confounding covariates were identified in relation to ARG presence, with the notable exception of antibiotic use within the last year (online supplemental table 2). A multivariable logistic regression analysis specific to the COPD group revealed a significant increase in tetracycline resistance mechanisms in patients with ≥2 exacerbations during the last year (online supplemental table 3). A list of the 212 identified ARGs is given in online supplemental table 4.

### ARG relative abundance

**Figure 2:**
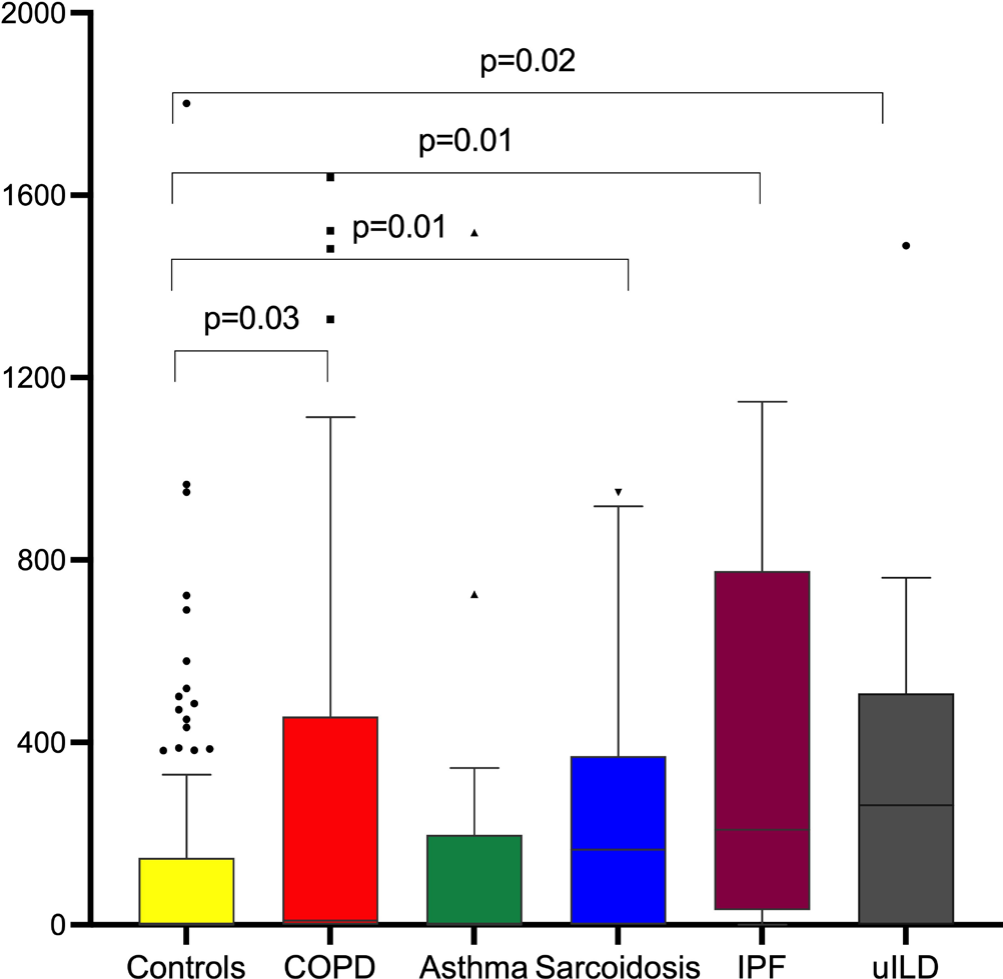
Mean counts per million of antibiotic resistance genes per bronchoalveolar lavage sample per study category. COPD, chronic obstructive pulmonary disease; IPF, idiopathic pulmonary fibrosis.

The counts per million for any ARG per BAL sample for the different patient groups compared with controls are shown in figure 2. Unadjusted, the counts per million were higher in patients with COPD, sarcoidosis, IPF and uILD compared with controls.

**Figure 3:**
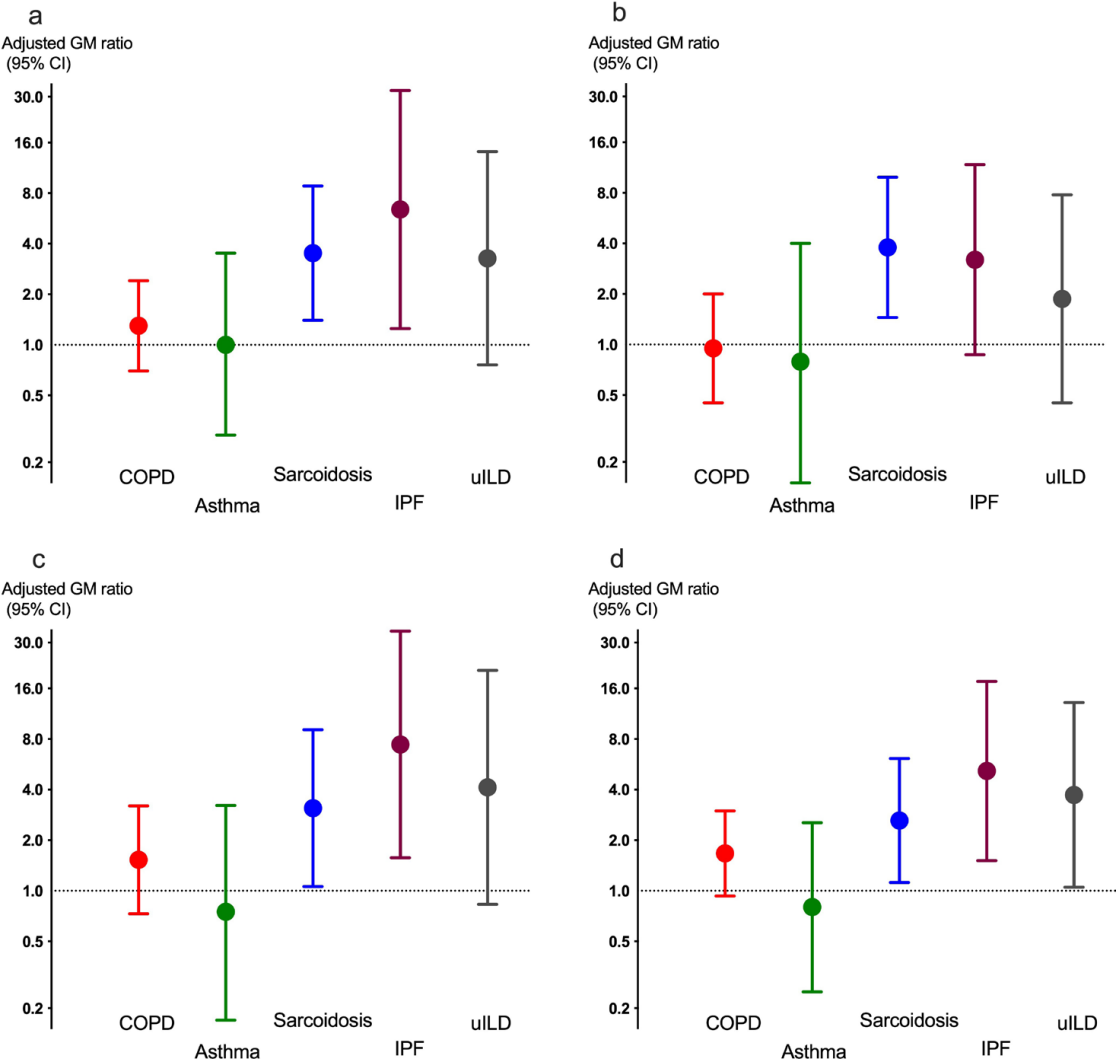
The GM ratio (95% CI) for normalised number of reads for ARGs for (a) all (b) beta-lactam ARGs (c) tetracycline ARGs, and (d) macrolide ARGs found in the respiratory microbiome in patients from each of the four diagnoses compared with healthy adults, adjusted for sex, age, body composition and smoking. ARGs, antimicrobial resistance gene; COPD, chronic obstructive pulmonary disease; GM, geometric mean; IPF, idiopathic pulmonary fibrosis.

The counts per million of the ARGs were non-normally distributed and thus log-transformed before running multivariable regression analysis for each of the five diagnoses compared with controls. The sarcoidosis group had significantly increased ARG counts per million, both overall and across the ARG classes beta-lactams, tetracyclines and macrolides compared with healthy controls after adjustment for sex, age, body composition and smoking (figure 3). Patients with IPF had increased counts per million of ARGs in the tetracycline and macrolide ARG classes. A trend towards a higher number of ARG counts per million in the macrolide class was observed in the COPD group, though not statistically significant.

### Previous antibiotic use

**Figure 4:**
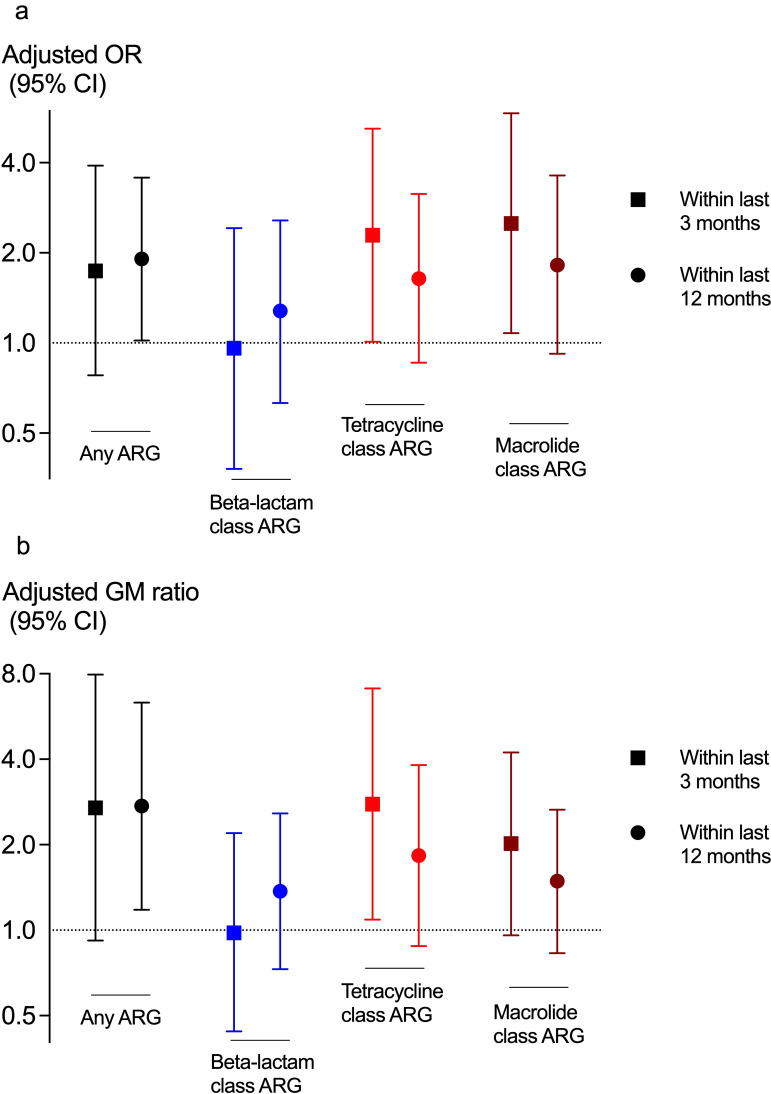
Impact of previous antibiotic use within the last 3 months or last year on the presence (a: OR, 95% CI) and normalised number of reads (b: GM ratio, 95% CI) of ARG in the respiratory microbiome, after adjustment for study category, sex, age, smoking and body composition. ARGs, antimicrobial resistance gene; GM, geometric mean.

Figure 4 shows the impact of antibiotic use within the past 3 months and the past year on presence of having any ARG (ORs) and relative abundance of ARGs (GM ratios), adjusted for study category and covariates. For most analyses, the trend favoured higher chances of having ARGs with previous antibiotic use within the last 3 or 12 months. This reached statistical significance for 4 out of 16 associations tested (figure 4).

## Discussion

This study demonstrates that both the presence and number of ARGs in the lower airways microbiome were significantly higher in patients with sarcoidosis and IPF compared with controls. A trend towards increased presence of ARGs was also observed in patients with COPD and uILD, but the difference compared with controls was not statistically significant. Both the presence/absence of ARGs and ARG counts per million were associated with antibiotic use within the last year.

Chronic lung diseases like COPD, asthma and ILDs all exhibit signs of inflammation in the airways and/or lung parenchyma. These diseases often exacerbate, most times due to airway infections and antibiotics are often deemed necessary. Yet, the reservoir of ARGs in the respiratory microbiome in health and disease is understudied, as are the environmental factors influencing the presence and size of the ARG reservoir. In a study from Ramsheh *et al,* 63 patients with COPD and 36 controls from three cohorts were sampled with either sputum (84) or bronchial brush (15), and 279 specific ARGs were quantified with quantitative PCR.^19^ More ARGs were detectable in sputum from patients with COPD, but no association was found between prior antibiotic use and ARG presence. However, data on antibiotic use was only available for 29 individuals, which was likely too low a number to draw inferences. In another study, 83 sputum samples were collected from 11 patients with asthma, 15 patients with COPD and 15 patients with bronchiectasis at two hospitals in Singapore, and compared with sputum samples from 13 healthy controls participating in a university-based exercise programme.^14^ Higher abundances of biosynthetic pathways associated with antibiotic consumption were seen in patients with COPD and bronchiectasis, particularly in the latter group. Only a few patients with COPD or bronchiectasis reported antibiotic use within the last 6 months, too few to make adjusted analyses. Apart from these two studies, there are notable studies on patients with bronchiectasis^17 18^ and patients in intensive care units.^15 16^ To our knowledge, no other studies on COPD or asthma are available, and no studies on ILDs and AMR. We also found no studies on these patient categories exploring the presence of ARGs using sterile sampling of the lower airways.

Although not statistically significant, the results from our study showed a higher proportion of ARGs in patients with COPD (50%) compared with healthy controls (38%), in line with the findings from Ramsheh *et al*^19^ and Mac Aogáin *et al*.^14^ As in the study by Mac Aogáin *et al* including 11 patients with asthma, our 13 patients with asthma did not have more ARGs than controls. A strength of our study is the consistent results obtained from analysing ARGs both as a binary outcome (presence/absence) and as counts per million. In both types of analysis, and both before and after adjustment for covariables, patients with sarcoidosis and IPF had more ARGs than the other groups.

We hypothesised that patients with chronic lung diseases, given their increased susceptibility to infectious exacerbations often treated with antibiotics, would exhibit a high prevalence of ARGs. In addition, we asked ourselves whether factors like active smoking and reduced lung function could impact the ARG presence, for instance by facilitating a load of less benign bacteria difficult to clear from the airways. Patients with COPD had a higher burden of ARGs only in the unadjusted analyses. Within our study sample, patients with COPD, IPF and uILD had the lowest lung function, and COPD and controls had the highest percentage of people who smoke. Antibiotic use in the past year was lowest among patients with sarcoidosis, even compared with controls, while it was higher in COPD, asthma, IPF and uILD. Only antibiotic use was convincingly related to presence and burden of ARGs among factors examined in our study. The patients with IPF and uILD were examined as part of a diagnostic work-up, in contrast to the other study groups. That is likely to explain why more of them had used antibiotics in the last 3 months and may be a factor in explaining the higher burden of ARGs compared with in COPD. In addition, we sampled the middle lobe, since it is the lobe where BAL yield is most consistent. However, the middle lobe may be more prone to harbour bacteria from micro-aspiration. Patients with IPF have a high prevalence of reflux, and this may mean they are more likely to have ARGs with a gastro-intestinal origin than, for instance, patients with COPD who have less reflux.

In Norway, penicillin is the first-line treatment for airways infections, also in patients with COPD, asthma and ILD. Although patients with sarcoidosis and IPF had microbiomes with increased beta-lactamase, tetracycline and macrolide class resistance mechanisms, only the latter two were significantly related to antibiotic use in adjusted analyses. Tetracyclines and macrolides may induce more resistance than penicillins and the functionality of an individual’s respiratory microbiome likely reflects both individual and environmental factors, which may explain our findings. However, antibiotic use was self-reported, and we lacked details on specific antibiotics taken. There was a trend towards a stronger relationship between ARGs and antibiotic use in the last 3 months compared with the last year. This may be because ARG presence is driven by selective pressure, and with more time from exposure the benefit from having ARGs wanes.

For patients with COPD, the sample size allowed for some adjustment for typical disease characteristics. There was no association between lung function or use of inhaled steroids and ARGs. However, frequent exacerbators were more likely to have tetracycline ARGs, even after adjusting for antibiotic use in the last year. The same trend was seen for macrolide ARGs, where patients with two or more exacerbations in the last year had significantly higher odds for having macrolide ARGs in a model excluding antibiotic use. No statistical interaction between exacerbations and antibiotic use was found in our study, but it is clinically highly plausible that frequent exacerbators see more antibiotics and thus acquire a microbiome with more ARGs.

A striking finding was the high prevalence of ARGs in patients with stable, untreated sarcoidosis, the group with the lowest exposure to antibiotics in the last year. No factor among those we examined explained this finding, but a sample size of 34 precluded extensive adjustment for covariables. Sarcoidosis is a granulomatous disease, where the immune system appears to mimic a defence like that against systemic fungal disease. Why this happens is still a mystery, and searches for a microbial cause of sarcoidosis have been futile. In another analysis from the MicroILD study, we have shown a significantly distorted mycobiome in patients with sarcoidosis compared with controls.^11^ Given that ARGs also arise in a diverse microbiome because of intramicrobial competition, one might speculate that patients with sarcoidosis have a dysbiosis in their fungal-bacterial microbiome, independent of the influence of antibiotic use.

In our study sample, several different resistance mechanisms were found and classified according to current databases. Multidrug-resistance efflux pumps of the ATP binding cassette and major facilitator superfamily were the most common resistance mechanisms found, in line with findings from the nasal resistome in children with cystic fibrosis.^30^ Classification of gene functionality in the microbiomes is a developing field, and classifications may change in the future. We provide a full list of the functional genes in the current study, for future reference and validation in future studies (online supplemental table 4).

There are some methodological issues to address. First, this is a cross-sectional study, which makes causal inferences impossible. Ideally, one would have prospective data on types and length of antibiotics used, but such data were unavailable in the current study. However, even with our imperfect study design, we observed a clear indication that antibiotic use impacts the presence of ARGs in the microbiome, and that the associations differed by disease category. Second, the sample size was quite low for patients with asthma, IPF and uILD in our study sample, precluding detailed subgroup analyses. Third, we employed protected sterile inner catheters for BAL sampling. This approach stands in contrast to most previous microbiome studies, where sampling has predominantly been performed using induced sputum, a method that is naturally more prone to upper airway contamination. Fourth, laboratory reagents like DNA extraction kits can carry some DNA contamination. Sequencing data from negative laboratory controls was not available, as DNA yields in these samples were consistently way below the threshold required for sequencing. This is consistent with minimal reagent contamination. However, obtaining BAL from a single lung lobe may not provide a comprehensive view of the pulmonary microbiome. Finally, mNGS data analysis relies on dynamic and incomplete databases, as this field of science is still evolving. The high proportion of human DNA obtained with BAL sampling from the respiratory tract combined with the relatively low abundance of ARGs necessitates sufficient sequencing depth.^31^ Recommended biostatistical methods to remove human DNA were employed in our analysis; however, it is possible that some ARGs were inadvertently excluded during this process.

Previous studies have shown that the composition of the respiratory microbiome differs between disease and health. This study extends this knowledge by showing that the functionality of the microbiome also differs between disease and health. Further, it underscores that antibiotic use impacts the presence of antibiotic resistance mechanisms in our microbiomes.

## Supplementary material

10.1136/bmjresp-2025-003864 online supplemental figure 1

10.1136/bmjresp-2025-003864 online supplemental table 1

10.1136/bmjresp-2025-003864 online supplemental table 2

10.1136/bmjresp-2025-003864 online supplemental table 3

10.1136/bmjresp-2025-003864 online supplemental table 4

10.1136/bmjresp-2025-003864 online supplemental file 1

## Data Availability

Data are available in a public, open access repository.
