# Supplementary figures and images for: Antimicrobial resistance genes and antibiotic use in chronic lung disease: a bronchoscopy study of the lower airways microbiome
Source: BMJ Open Respir Res. 2026 Mar 25;13(1):e003864. doi: 10.1136/bmjresp-2025-003864 (PMC13034347; doi:10.1136/bmjresp-2025-003864)

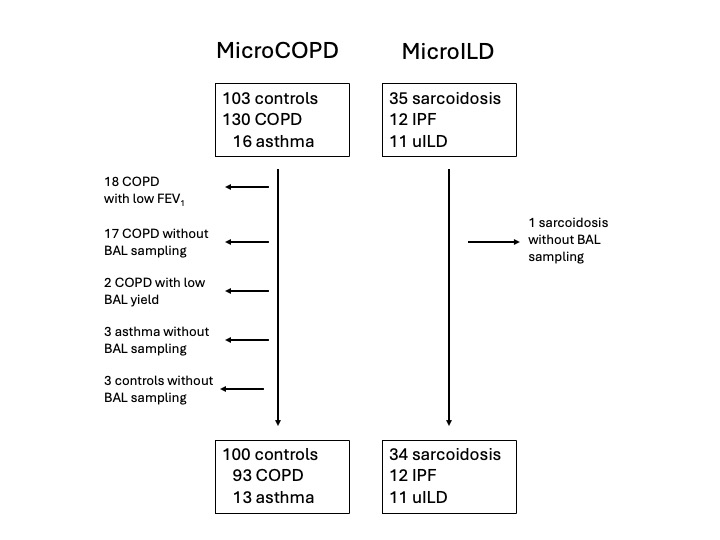

Supplement: online supplemental figure 1 [file bmjresp-13-1-s001.jpg]
