# Supplementary material for: Antimicrobial resistance genes and antibiotic use in chronic lung disease: a bronchoscopy study of the lower airways microbiome
Source: BMJ Open Respir Res. 2026 Mar 25;13(1):e003864. doi: 10.1136/bmjresp-2025-003864 (PMC13034347; doi:10.1136/bmjresp-2025-003864)
Supplement: online supplemental file 1 [file bmjresp-13-1-s006.docx]

# Comparison between NDARO and CARD AMR databases

## Methods

To evaluate how comprehensively NDARO represents the AMR protein repertoire curated in CARD, we retrieved the protein FASTA datasets from both resources by using the “Homolog model” and “Overexpression model” files for CARD, and filtering NDARO to exclude entries classified as “VIRULENCE”, “STRESS”, or “POINT” in order to retain only acquired resistance proteins. This focus on acquired genes was chosen because, in metagenomic datasets, these genes are generally complete, mobile, and interpretable, whereas point-mutation–based resistance is more difficult to distinguish from sequencing errors at the low sequencing depth after human decontamination of our datasets.

We then performed an all-versus-all DIAMOND (v2.1.8) blastp comparison, aligning NDARO proteins against CARD under three increasingly stringent thresholds (90%, 95%, and 99% identity with bidirectional coverage of at least 90%). All analyses were executed within a controlled Docker environment to ensure full reproducibility, and for each threshold we quantified the proportion of CARD proteins with at least one matching NDARO sequence, providing a direct measure of CARD coverage.

## Results

From the CARD database, we retained only entries labelled as “Homolog model” or “Overexpression model”, resulting in a set of 6,067 proteins. Filtering the NDARO database to exclude proteins classified as “VIRULENCE”, ”STRESS”, or “POINT” yielded a final set of 8,115 proteins.

Across all stringency levels, NDARO consistently recovered the vast majority of CARD AMR proteins. At 90% identity and coverage, 5,724 out of 6,067 CARD proteins were matched by NDARO (94.35%). Coverage remained nearly unchanged at 95% (5,675 proteins; 93.54%) and even under the strict 99% cutoff (5,657 proteins; 93.24%) (table 1). Overall, NDARO contains more than 93% of the CARD protein set across all thresholds, demonstrating strong concordance between the two databases.

**Table 1**. Performance metrics obtained using progressively more stringent thresholds.

| Value | Minimum identity | | |
| --- | --- | --- | --- |
|  | **90%** | **95%** | **99%** |
| NDARO proteins with ≥1 CARD hit | 7,614 (93.83%) | 7,431 (91.57%) | 6,798 (83.77%) |
| CARD proteins with ≥1 NDARO hit | 5,724 (94.35%) | 5,675 (93.54%) | 5,657 (93.24%) |
| Average identity | 98.35% | 98.75% | 99.43% |
| Average alignment length | 316.00 aa | 317.78 aa | 328.75 aa |
